# The Role of Alternative Splicing in Lung Cancer: Mechanisms, Regulators, and Therapeutic Implications

**DOI:** 10.3390/ijms27167269

**Published:** 2026-08-14

**Authors:** Lingrui Shang, Nannan Wang, Qianqian Liu, Lihua Chen, Huisheng Liu

**Affiliations:** 1 School of Biomedical Engineering, Guangzhou Medical University, Guangzhou 511436, China; shang_lingrui@gzlab.ac.cn (L.S.); liu_qianqian@gzlab.ac.cn (Q.L.); 2 Department of Diagnostics and Detection Technologies, Guangzhou National Laboratory, Guangzhou 510005, China; wang_nannan@gzlab.ac.cn; 3 College of Life Science & Technology, Huazhong University of Science and Technology, Wuhan 430074, China; 4 School of Forestry and Biotechnology, Zhejiang A&F University, Hangzhou 311300, China; 5 State Key Laboratory for Development and Utilization of Forest Food Resources, Zhejiang A&F University, Hangzhou 311300, China

**Keywords:** lung cancer, alternative splicing, CD44

## Abstract

Lung cancer is characterized by profound molecular and clinical heterogeneity and remains the leading cause of cancer-related mortality worldwide. Its initiation and progression are shaped by complex genetic and epigenetic alterations that perturb key biological processes, including cell proliferation, apoptosis, metabolic reprogramming, invasion, metastasis, and immune evasion. Increasing evidence indicates that dysregulated alternative splicing (AS) represents a critical post-transcriptional regulatory mechanism involved in lung cancer pathogenesis. Although AS abnormalities are not the sole drivers of tumorigenesis, they contribute to malignant transformation, tumor progression, therapeutic resistance, and phenotypic plasticity, providing opportunities for biomarker development and therapeutic targeting. This review summarizes the multifaceted roles of AS in lung cancer biology, highlighting its contributions to tumor evolution, metastatic dissemination, and treatment resistance. Furthermore, subtype-specific AS landscapes between major lung cancer subtypes are discussed to elucidate their distinct molecular mechanisms and therapeutic implications. Representative AS-generated isoforms, including cluster of differentiation 44 variants (CD44v), are further discussed as examples of how aberrant splicing events regulate cancer stemness, tumor progression, and therapeutic responses. The regulatory mechanisms underlying CD44v generation, including upstream splicing factors and downstream signaling pathways, are also summarized. Collectively, this review highlights the emerging role of aberrant AS regulation in lung cancer and emphasizes its potential implications for biomarker discovery and precision therapeutic strategies targeting splicing dysregulation.

## 1. Introduction

Carcinogenesis is a complex process involving the interplay of multiple factors and mechanisms [1], including genetic variations, aberrant signaling pathways, and alterations in the microenvironment [2,3]. Tumor cells typically exhibit significant genetic heterogeneity, with mutations that activate or inhibit key genes and signaling pathways. Additionally, tumor cells utilize mechanisms such as metabolic reprogramming, immune evasion, and angiogenesis to survive and disseminate within the host [4,5,6]. Lung cancer is the most prevalent malignant tumor worldwide, with the highest incidence and mortality rates [7]. This tumor type exhibits pronounced heterogeneity, evident in the diversity among patients regarding their genetic mutation profiles, molecular characteristics, cellular composition, proliferation rates, metastatic potential, and treatment responses. Such variations contribute to the challenges in diagnosing and treating the tumor, as they can influence both the clinical presentation and the therapeutic outcomes. Understanding these diverse factors is crucial for developing personalized treatment strategies and improving patient prognosis [8,9]. Lung cancer initiation is often associated with dysregulation of cell cycle control, aberrant activation of signaling pathways, and the accumulation of mutations in pivotal genes. Moreover, changes in the tumor microenvironment, such as immune suppression, hypoxia, and matrix remodeling, promote lung cancer progression [10,11]. However, many aspects of lung cancer, particularly those related to extracellular matrix regulation, intercellular communication, and immune evasion, remain poorly understood.

**Figure 1:**
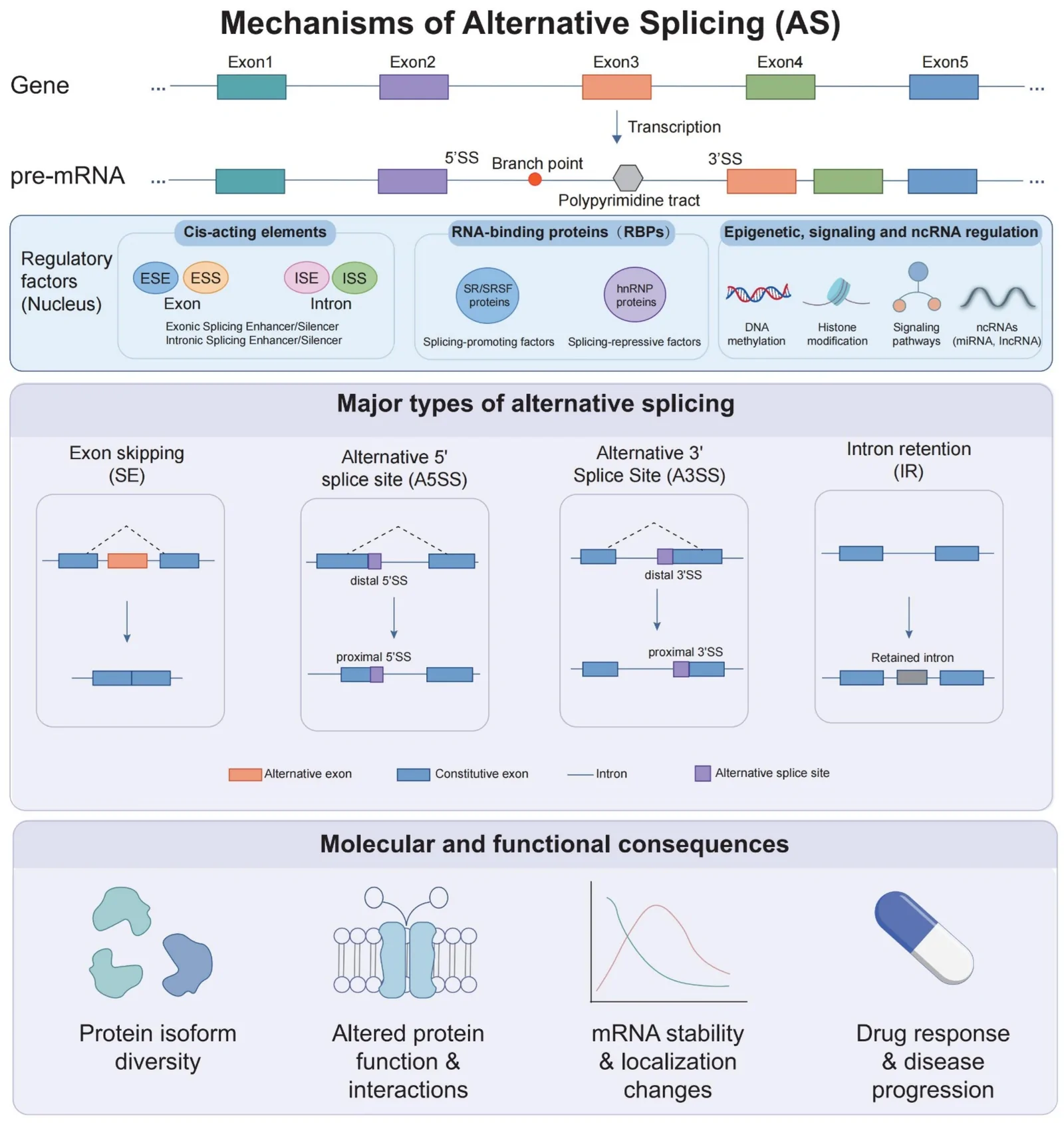
Regulatory mechanisms and biological consequences of alternative splicing. Alternative splicing (AS) is a fundamental post-transcriptional regulatory process that expands transcriptomic and proteomic diversity by producing multiple mRNA isoforms from a single pre-mRNA. Major AS patterns include exon skipping (SE), alternative 5′ splice site selection (A5SS), alternative 3′ splice site selection (A3SS), and intron retention (IR). AS regulation is mediated through coordinated interactions among cis-regulatory elements, RNA-binding proteins, and epigenetic, signaling, and non-coding RNA networks, which determine splice-site selection and spliceosome activity. Aberrant AS alters protein isoform composition, molecular interactions, and mRNA properties, thereby influencing cellular functions, disease progression, and responses to therapeutic interventions.

AS, a fundamental post-transcriptional regulatory mechanism, profoundly diversifies the transcriptome and expands proteomic complexity by generating multiple distinct mRNA isoforms from a single precursor mRNA. Tumors exhibit more AS events than normal tissues, generating cancer-specific splice isoforms [12,13,14]. The fundamental mechanisms of AS regulation, including splice site selection, exon inclusion or exclusion, and the generation of functionally distinct transcript isoforms, are illustrated in Figure 1. Emerging evidence has revealed that AS plays a crucial regulatory role in the initiation and progression of lung cancer [15,16]. Recent studies have revealed that numerous abnormal splicing events prevalent in lung cancer involve critical oncogenes and tumor suppressor genes, including B-cell lymphoma 2 Like 1 (*BCL2L1*), Mdm2 p53 binding protein homolog (*MDM2*), Mdm4 p53 binding protein homolog (*MDM4*), endocytic adaptor protein (*NUMB*), and mesenchymal–epithelial transition factor (*MET*). These splicing alterations contribute to tumorigenesis by modulating critical processes, including apoptosis, cell cycle regulation, signal transduction, and drug resistance [17]. Large-scale transcriptomic analyses have revealed extensive aberrant alternative splicing events in lung cancer, many of which remain functionally uncharacterized and require further investigation. These results highlight the potential significance of aberrant AS events in lung cancer heterogeneity and the variability in therapeutic responses [18]. Therefore, in-depth investigations into its mechanisms and regulatory networks are expected to drive advancements in lung cancer diagnosis and treatment. These advancements include the development of novel strategies in precision medicine, targeted therapies, and immunotherapies, which could lead to more effective clinical interventions.

Research has shown that the crucial cell surface glycoprotein CD44 is particularly elevated in lung cancer [19] where it is strongly associated with increased invasiveness and metastatic potential [20]. Through its interactions with the extracellular matrix, *CD44* modulates tumor cell migration and metastasis, while also interacting with immune cells to promote tumor immune evasion [21]. CD44 variants (CD44v) are generated through regulated alternative splicing of the CD44 transcript, involving the selective inclusion of variable exons (v2–v10) between conserved constant regions. These distinct exon combinations generate multiple CD44v isoforms with different extracellular domains and functional properties, contributing to diverse roles in tumor progression [22,23,24], which play diverse roles in various tumors. The functional diversity of CD44v positions it as a key target for understanding cancer mechanisms and developing targeted therapies [25,26]. Despite this, specific mechanisms of CD44v in lung cancer remain understudied. The functional diversity of CD44v underscores its role as a key target for cancer research and targeted therapy development [27,28,29]. However, the roles of CD44v across different lung cancer subtypes are not fully understood. Consequently, there is a pressing need to elucidate the molecular mechanisms underlying CD44v’s role in lung cancer pathogenesis and progression, as well as to explore its potential as a therapeutic target.

This review provides a systematic summary of the function of AS in lung cancers, encompassing the development, progression, invasion, metastasis and drug resistance of the disease. Furthermore, it summarizes recent advancements in understanding the biological functions of CD44 and its variant forms CD44v in cancer, with a particular emphasis on lung cancer. This work not only offers a new theoretical foundation for understanding the pathogenesis of lung cancer but also establishes a crucial molecular basis for the development of targeted therapies directed against AS in lung cancer.

## 2. Alternative Splicing in Lung Cancer

**Figure 2:**
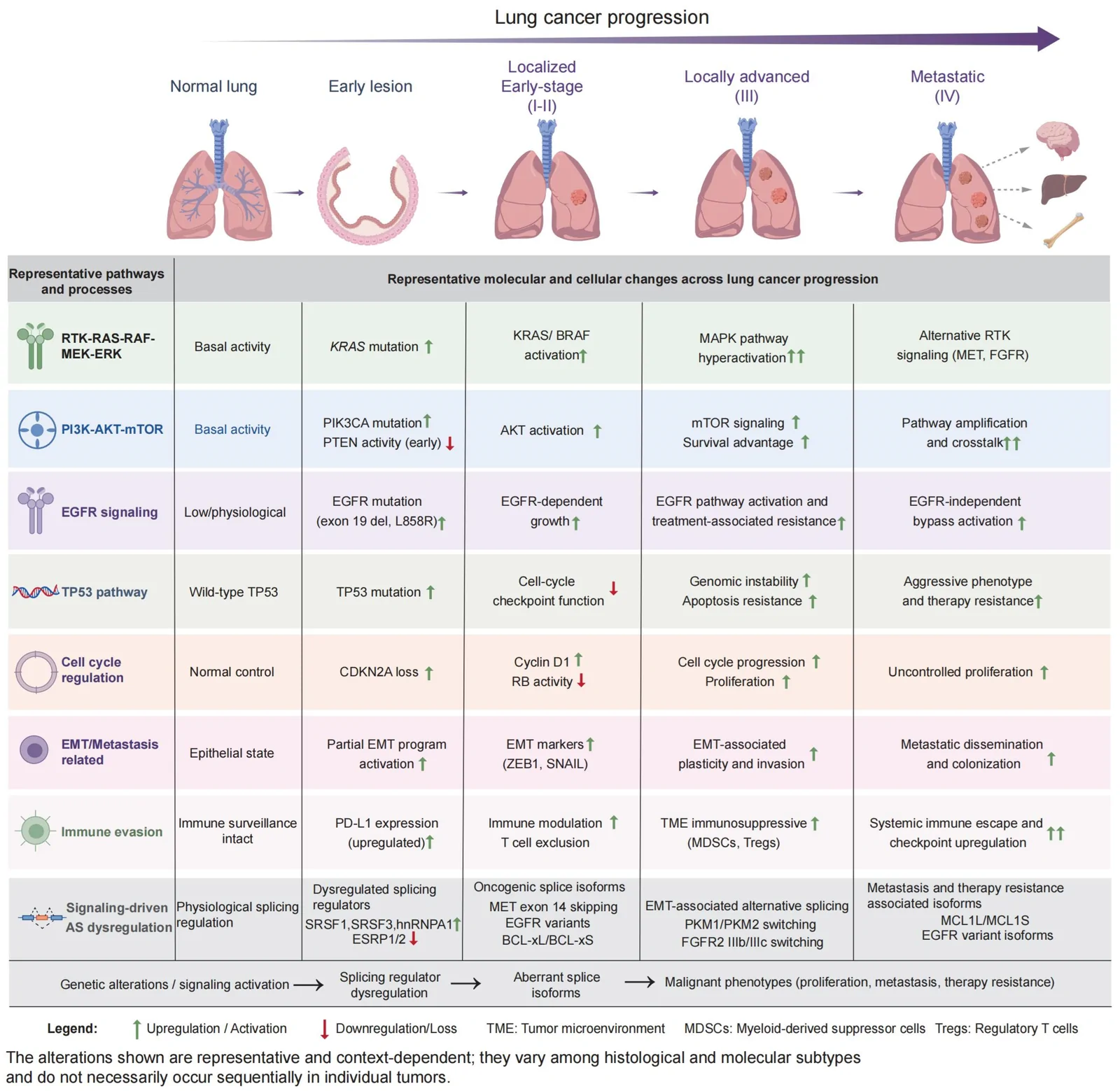
Key molecular alterations and alternative splicing dysregulation driving lung cancer progression. Overview of representative genetic, signaling, cellular, and alternative splicing alterations during lung cancer evolution from normal lung tissue to metastatic disease. The figure highlights oncogenic pathway activation, dysregulation of splicing regulators, generation of aberrant splice isoforms, and their roles in promoting malignant phenotypes, including proliferation, EMT-associated metastasis, immune escape, and therapy resistance.

During lung cancer development and progression, sequential accumulation of genetic alterations and activation of oncogenic signaling pathways drive malignant transformation, metastasis, and therapeutic resistance (Figure 2). These molecular changes not only determine tumor phenotypes but also profoundly influence post-transcriptional regulation by reshaping the activity of splicing regulators and the alternative splicing (AS) landscape. Aberrant activation of oncogenic signaling pathways can profoundly reshape the alternative splicing (AS) landscape in lung cancer by altering the activity of key splicing regulators. Among these oncogenic regulators, serine and arginine rich splicing factor 1 (SRSF1) represents a well-established oncogenic splicing factor that is frequently upregulated in lung adenocarcinoma (LUAD) and lung squamous cell carcinoma (LUSC). SRSF1 promotes tumor cell survival and proliferation through regulation of cancer-associated AS events and activation of oncogenic signaling pathways, including mTOR-related signaling [30]. In addition, oncogenic KRAS signaling contributes to widespread AS dysregulation in LUAD by suppressing serine/arginine-rich protein (SR protein) phosphorylation, thereby impairing the activity of key splicing factors. This regulation predominantly induces exon-skipping events independent of changes in splicing factor expression. Multiple conserved KRAS-driven AS events have been identified, including MYC associated zinc finger protein (MAZ) isoforms that regulate KRAS expression, suggesting the presence of oncogenic feedback loops that further reinforce KRAS signaling and splicing dysregulation [16]. These findings demonstrate that oncogenic signaling and splicing regulation are interconnected networks that promote lung cancer initiation and progression.

In addition to oncogenic splicing regulators, tumor-associated RNA-binding proteins (RBPs) contribute substantially to the remodeling of the AS landscape in lung cancer. RNA binding motif protein 5 (RBM5), RNA binding motif protein 6 (RBM6), and RNA binding motif protein 10 (RBM10) are frequently altered in lung cancer, and their deletion or mutation disrupts normal splicing homeostasis, leading to aberrant regulation of multiple downstream targets [15]. A representative example is NUMB exon 9 splicing, which determines Notch signaling activity and tumorigenic potential. Exon 9 skipping generates a NUMB isoform that suppresses Notch signaling and exerts tumor-suppressive effects, whereas exon 9 inclusion promotes Notch activation and tumor progression. Notably, RBM5/6 and RBM10 exert differential regulatory effects on NUMB splicing, while RBM10 mutations further enhance aberrant cellular proliferation, highlighting the importance of RBM-mediated splicing regulation in lung cancer progression. Similarly, quaking, KH domain containing RNA binding (QKI), functions as an important tumor-associated splicing regulator in non-small cell lung cancer (NSCLC). Reduced QKI expression is associated with tumor progression and poor prognosis [31]. Through regulation of multiple AS events, particularly those involved in cytoskeletal remodeling and endocytic processes, such as extended synaptotagmin 2 (ESYT2), QKI influences tumor cell morphology, migration, and cellular dynamics. Distinct ESYT2 splice variants generated by QKI-dependent regulation exhibit different biological effects on cytoskeletal organization and endocytosis, emphasizing the contribution of tumor-associated splicing regulators to lung cancer heterogeneity.

In contrast to oncogenic splicing regulators, tumor-suppressive splicing factors maintain cellular homeostasis by promoting anti-tumorigenic splice isoforms. RBM4 is a representative tumor-suppressive splicing factor that regulates cancer-associated AS events in lung cancer cells [32,33]. RBM4 expression suppresses tumor cell proliferation and migration by modulating BCL-X alternative splicing, promoting production of the pro-apoptotic BCL-XS isoform while reducing expression of the anti-apoptotic BCL-XL isoform. This shift in splice isoform balance enhances apoptotic signaling and inhibits tumor growth. Collectively, these findings demonstrate that the functional balance between oncogenic and tumor-suppressive splicing factors determines the AS landscape of lung cancer and contributes to tumor development, progression, and therapeutic response [34,35].

## 3. The Differences in AS Between LUAD and LUSC

LUAD and LUSC are the two primary subtypes of NSCLC, displaying significant differences in pathogenesis, histological origin, biological behavior, molecular profiles, and clinical outcomes. Their driver mutation landscapes are markedly distinct [9]. LUAD is commonly associated with mutations in *EGFR*, *KRAS*, *ALK* receptor tyrosine kinase (*ALK*), ROS proto-oncogene 1, receptor tyrosine kinase (*ROS1*), B-Raf proto-oncogene, serine/threonine kinase (*BRAF*), and *MET* [36]. In contrast, LUSC predominantly harbors *TP53* mutations, which is often accompanied by phosphatidylinositol-4,5-bisphosphate 3-kinase catalytic subunit alpha (*PIK3CA*) mutations, fibroblast growth factor receptor 1 (*FGFR1*) amplification, SRY-box transcription factor 2 (*SOX2*) amplification, and cyclin dependent kinase inhibitor 2A (*CDKN2A*) deletion [7,37,38]. The oncogenic pathways in LUAD are primarily linked to the EGFR signaling cascade [39], PI3K-AKT, and MAPK pathways, whereas LUSC is more associated with oxidative stress, dysregulated Notch signaling, and aberrant cell cycle control [40]. To summarize the distinct molecular characteristics and biological features of LUAD and LUSC, the major differences in their driver alterations, associated signaling pathways, and alternative splicing patterns are summarized in Table 1.

In LUAD, AS predominantly affects driver genes and their associated signaling pathways, which in turn regulate tumor cell proliferation, metabolic adaptation, and therapeutic sensitivity [12,16,41]. Studies have demonstrated that in LUAD, alternative splicing events affecting receptor tyrosine kinase signaling pathways, including EGFR, have been implicated in modulating therapeutic sensitivity to tyrosine kinase inhibitors [16,20], thereby reducing treatment response and shortening patient survival. Similarly, RAS related 2 (*RRAS2*) generates multiple splicing isoforms, including ES and alternative termination (AT) variants, with distinct functional isoforms that modulate downstream Ras signaling pathways, influencing cell differentiation and tumor progression [2]. In contrast, AS events in LUSC are more concentrated in genes involved in cell adhesion, immune regulation, and oxidative stress responses [9,30]. AS isoforms of certain genes exert distinct biological functions across different lung cancer. For instance, fibroblast growth factor receptor 2 (FGFR2) in LUSC produces the IIIc splice variant, which, while consistent with LUAD expression patterns, may preferentially regulate cellular responses to external growth factor stimuli, thereby enhancing proliferative signals [42]. Although the precise splicing variants of *SOX2* remain undefined, its overexpression in LUSC is closely linked to squamous epithelial differentiation and the maintenance of stemness, suggesting the existence of functional splicing isoforms [43]. Emerging evidence suggests that alternative splicing may contribute to functional diversity between LUAD and LUSC by generating distinct transcript isoforms with different biological properties. For example, FGFR2 IIIb and IIIc isoforms differ in their extracellular ligand-binding domains, resulting in altered growth factor responsiveness and potentially contributing to subtype-specific tumor behaviors [42]. Although direct comparisons of identical splice isoforms between LUAD and LUSC remain limited, accumulating evidence indicates that subtype-specific splicing programs contribute to the molecular heterogeneity and distinct biological characteristics of NSCLC.

Notably, certain splicing factors exhibit opposite biological effects in LUSC compared to LUAD. In LUAD, AS predominantly affects mechanisms such as driver gene mutations, signaling pathway activation, and metabolic adaptation. In contrast, in LUSC, splicing events are more closely linked to maintaining cellular architecture, immune regulation, and epithelial differentiation [38,44].

Splicing quantitative trait loci (sQTLs) are specific genetic variants within the genome that influence pre-mRNA splicing [45]. These variants modulate gene expression by altering splicing patterns, potentially affecting phenotypic traits and disease susceptibility. In the context of lung cancer, sQTLs have been shown to significantly influence the risk of LUAD, whereas their impact on squamous cell carcinoma appears to be limited. Specifically, in LUAD, splicing alterations in multiple genes are significantly associated with disease risk. These aberrant splicing events may contribute to the pathogenesis of LUAD by modifying transcript isoform profiles. In contrast, sQTLs in squamous cell carcinoma show no significant association with disease risk. Further analyses reveal that sQTLs associated with LUAD and genetic variants linked to squamous cell carcinoma exhibit distinct gene-specific splicing patterns, suggesting that these lung cancer subtypes may be driven by divergent molecular mechanisms. Thus, sQTLs not only play a pivotal role in LUAD development but also offer critical insights into the distinct molecular mechanisms underlying different lung cancer subtypes [46,47].

In conclusion, an in-depth exploration of subtype-specific AS events and their regulatory factors in NSCLC may facilitate the development of novel biomarkers and molecular-targeted therapies, providing a theoretical foundation for more precise classification, diagnosis, and treatment of lung cancer.

## 4. Regulation of Alternative Splicing in Lung Cancer

AS regulatory factors, as the core mechanism of RNA processing, play a pivotal regulatory role in the occurrence, progression and heterogeneity of lung cancer. Regulatory factors mainly include RBPs, splicing factors, and epigenetic modification enzymes. These factors shape the tumor-specific microenvironment by dynamically regulating exon inclusion/skipping events. As a key RBP, QKI dysregulation in diverse cancers primarily manifests through the modulation of AS events, which promotes the inclusion of oncogenic isoforms in disease-associated genes, thereby driving cell proliferation and immune evasion. Research has found that QKI, a regulator of alternative splicing in lung cancer, can influence tumor progression through modulation of splicing programs in LUSC [18,30]. The SRSF family proteins regulate alternative splicing through mechanisms distinct from QKI. In lung cancer, SRSF1 has been implicated in promoting tumor cell proliferation by modulating alternative splicing programs associated with oncogenic signaling pathways, including MAPK-related signaling [17,48]. Recent studies have identified that poly (ADP-ribose) polymerase family member 4 (PARP4) and heterogeneous ribonucleoprotein M (hnRNPM) cooperatively regulate tumor-associated splicing programs, thereby contributing to LUAD progression [49]. Beyond hnRNPM, other RNA-binding proteins and spliceosomal components have also been implicated in lung cancer-associated splicing dysregulation. U2AF1, a core spliceosomal factor, is recurrently altered in LUAD, where the hotspot S34F mutation perturbs 3′ splice-site recognition [50] and remodels the alternative splicing landscape. In KRAS-mutant LUAD, the *U2AF1* S34F mutation confers a selective advantage by rescuing aberrant KRAS splicing defects, restoring MAPK signaling, and promoting tumor progression [51]. In addition, PTBP1 and HNRNPC represent important RNA-binding splicing regulators involved in distinct oncogenic processes. PTBP1 contributes to lung cancer progression by regulating both therapeutic resistance and metabolic adaptation, through sustaining TAK1 expression to suppress RIPK1-mediated PANoptosis in EGFR-TKI-resistant cells [52] and promoting *PKM* alternative splicing to enhance aerobic glycolysis [53]. HNRNPC promotes NSCLC progression by recognizing m6A-modified *TFAP2A* transcripts and maintaining their stability, thereby activating the TFAP2A/CTNNB1 axis and inducing EMT-associated malignant phenotypes [54]. Loss or mutation of PARP4—such as the clinically observed PARP4 depletion or mutation (I1039T) variant—significantly enhances the tumorigenic potential of KRAS- or EGFR-driven lung cancer cells. Moreover, depletion of its upstream regulatory factor hnRNPM results in widespread splicing defects, including intron retention in pre-mRNA processing factor 4B (*PRPF4B*) and transmembrane protein 107 (*TMEM107*) and single-exon skipping events. Together, these findings indicate that AS regulatory factors constitute a highly coordinated and dynamic regulatory network in lung cancer. Dysregulation of key RNA-binding proteins and splicing modulators, including QKI, PTBP1, HNRNPC, hnRNPM, SRSF family proteins, PARP4, and U2AF1, drives lung cancer progression by remodeling cancer-specific splicing landscapes, activating oncogenic signaling pathways, and promoting tumor heterogeneity, immune evasion, and therapeutic resistance.

**Figure 3:**
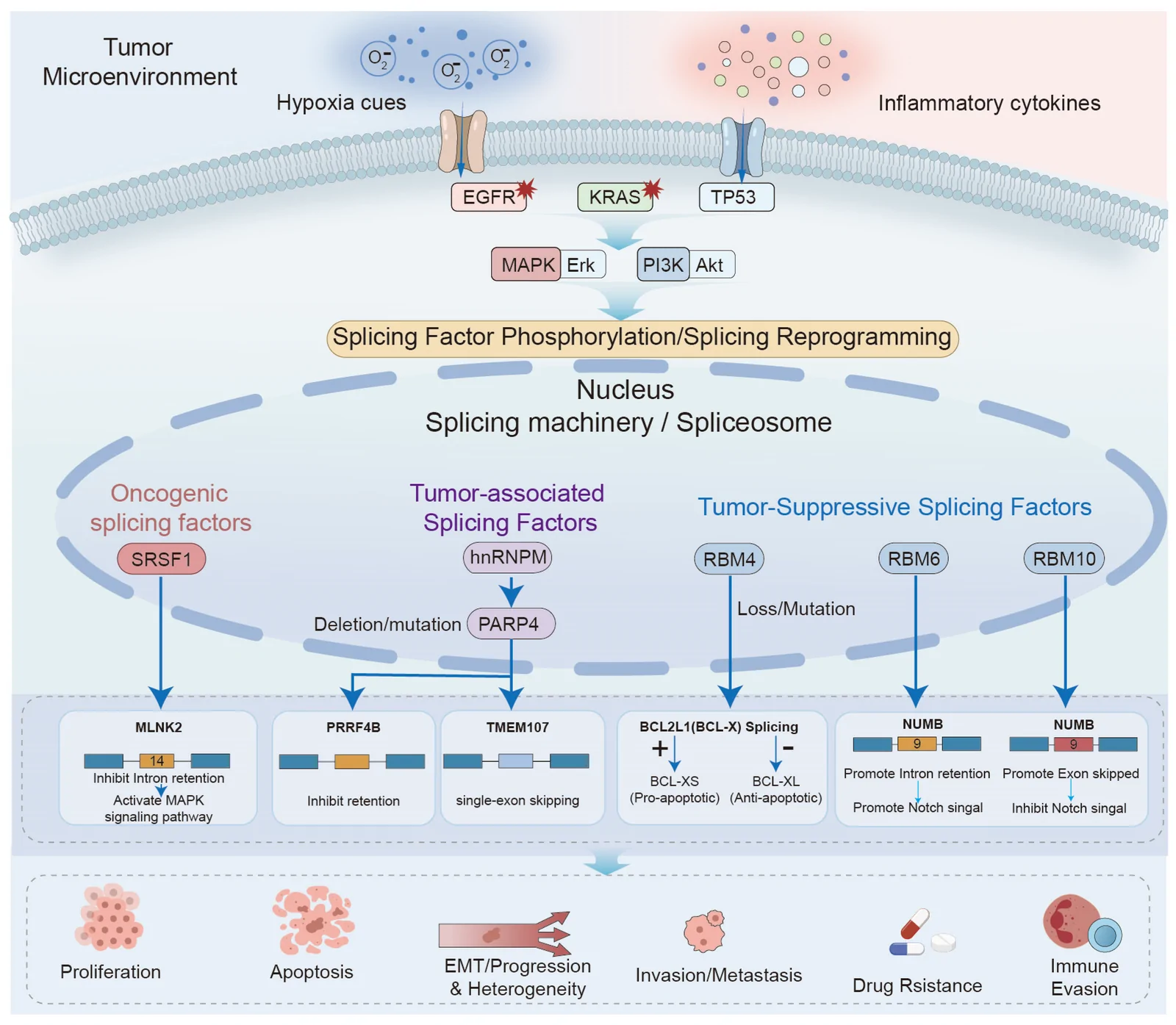
Dysregulated alternative splicing networks drive lung cancer progression and therapeutic resistance. Tumor microenvironment-mediated alternative splicing drives lung cancer progression. Tumor microenvironmental cues, including hypoxia, inflammatory cytokines, and oncogenic signaling, activate receptor-mediated pathways such as EGFR, KRAS, and TP53, leading to the activation of downstream MAPK–ERK and PI3K–AKT signaling cascades. These pathways induce phosphorylation of splicing factors and global splicing reprogramming through modulation of the spliceosome machinery. Dysregulated splicing factors are categorized into oncogenic splicing factors (e.g., SRSF1), tumor-associated splicing factors (e.g., hnRNPM and PARP4), and tumor-suppressive splicing factors (e.g., RBM4, RBM6, and RBM10), which are frequently altered by overexpression, deletion, or mutation in lung cancer. Aberrant alternative splicing events affect key cancer-related genes, including MLNK2, PRPF4B, TMEM107, BCL2L1, and NUMB, resulting in altered exon inclusion or skipping and shifts in pro- and anti-apoptotic isoforms. Collectively, these splicing alterations promote tumor cell proliferation, apoptosis resistance, epithelial–mesenchymal transition (EMT), invasion and metastasis, therapeutic resistance, and immune evasion, thereby driving lung cancer progression.

Collectively, these findings underscore the central importance of AS regulators in shaping lung cancer biology. By orchestrating distinct yet convergent splicing networks, RBPs such as *QKI* [31,55] (Figure 3), SRSF1, and PARP4—together with their cofactors—construct tumor-specific transcriptomic landscapes that promote malignant transformation, cellular plasticity, and therapeutic resistance (Figure 3). Elucidating these splicing-driven regulatory circuits will be essential for understanding lung cancer heterogeneity and may ultimately provide new avenues for biomarker development and precision-targeted therapies.

## 5. Dysregulation of Key Lung Cancer-Related Genes Alternative Splicing

Abnormal AS of lung cancer driver genes and tumor suppressor genes can alter protein function, activate oncogenic signals or lose tumor suppressor effects [56], thereby promoting the occurrence, progression, invasion, and drug resistance of lung cancer. *KRAS* is the most frequently mutated oncogenic driver in LUAD. Abnormal AS of lung cancer driver genes and tumor suppressor genes can alter protein function, activate oncogenic signals, or impair tumor suppressive effects [57], thereby promoting lung cancer initiation, progression, invasion, and drug resistance. KRAS is one of the most frequently mutated oncogenic drivers in LUAD. Constitutively activated KRAS signaling promotes tumor cell proliferation, survival, and metastatic dissemination primarily through the RAS–MAPK and PI3K–AKT pathways. Clinically, KRAS alterations are associated with therapeutic resistance, remodeling of the tumor immune microenvironment, and poor prognosis. Through alternative splicing of exon 4, KRAS generates two major isoforms, KRAS4A and KRAS4B, which differ in their C-terminal hypervariable regions and exhibit distinct subcellular localization and signaling properties. These isoforms display distinct biological activities and may differentially influence LUAD progression through modulation of oncogenic signaling pathways [16,47]. Beyond transcriptional dysregulation, oncogenic *KRAS* has also been shown to induce widespread changes in AS programs in lung epithelial cells. Mechanistically, KRAS pathway activation can alter the phosphorylation status of splicing regulators, particularly SR proteins, thereby driving post-transcriptional splicing reprogramming. Such KRAS-associated splicing alterations may contribute to lung tumorigenesis and help explain inter-tumoral heterogeneity in treatment response [2,11,16]. In addition to oncogenes, the tumor suppressor gene *TP53* is among the most frequently mutated genes in lung cancer. Under physiological conditions, MDM2 and MDM4 maintain cellular homeostasis by inhibiting p53 activity. However, accumulating evidence indicates that in lung cancer and several other malignancies, aberrant pre-mRNA splicing of MDM2 and MDM4 generates multiple isoforms that lack critical functional domains. In lung cancer, aberrant alternative splicing of MDM2 and MDM4 generates multiple isoforms with altered functional domains, which may disrupt p53 tumor suppressor activity by modulating the MDM2-p53 regulatory axis. Consequently, this aberrant isoform attenuates the tumor-suppressive activity of p53 and facilitates malignant progression [7,11,12].

CD44, a highly heterogeneous transmembrane glycoprotein [57,58], generates multiple functional isoforms through AS, playing a critical role in lung cancer development Selective splicing of the variable exon (v6–v10) region of CD44 is prevalent in both LUAD and LUSC. In LUAD, CD44v6 promotes EMT and enhances metastatic potential, significantly correlating with poor survival prognosis. Moreover, CD44 alternative splicing generates functional isoforms, such as CD44s and CD44v8-10, which contribute to acquired osimertinib resistance through activation of the ErbB3/STAT3 signaling pathway in LUAD [58]. Thus, AS of CD44 not only drives phenotypic plasticity in lung cancer cells but also holds promise as a prognostic biomarker and therapeutic target [59]. The *CD44* gene produces multiple splice variants through AS, each comprising distinct combinations of exons. AS of CD44 predominantly occurs within exon 6, exon 5A, and several other variable exon regions. The selective inclusion of these exons defines distinct CD44 isoforms, giving rise to variants such as CD44s, CD44v, CD44v3, and CD44v10, which exhibit substantial structural and functional diversity [60]. The upstream regulatory mechanisms of CD44v exhibit a multi-layered and highly intricate network architecture. First, several canonical signaling pathways (including Wnt/β-catenin, PI3K/AKT, and MAPK/ERK) regulate CD44 transcriptional activity and AS by modulating specific transcription factors, thereby dictating CD44v expression levels. Second, critical transcription factors (e.g., c-Myc, HIF-1α, and NF-κB) directly or indirectly promote CD44v transcription and potentiate its roles in tumorigenesis, invasion, and therapeutic resistance. In addition, AS regulators (e.g., ESRP1/2, hnRNP family, and SR proteins) play pivotal roles in exon selection and constitute essential determinants of distinct CD44v isoform formation. Regulation of CD44v by the tumor microenvironment represents a critical layer of control. Hypoxic conditions, pro-inflammatory cytokines (e.g., TGF-β, IL-6), and tumor-associated fibroblasts can indirectly upregulate CD44v expression through the activation of specific signaling cascades or transcriptional programs. The small nucleolar RNA, H/ACA box 37 (SNORA37)/cap methyltransferase 1 (CMTR1)/ELAV like RNA binding protein 1 (ELAVL1) feedback loop has been identified as a pivotal upstream regulator of CD44v AS in gastric cancer. SNORA37 enhances the CMTR1–ELAVL1 interaction, promoting the nuclear retention and splicing activity of ELAVL1, thereby driving the production of oncogenic CD44v6 while reducing CD44s [61]. Moreover, epigenetic modifications—including DNA methylation, histone remodeling, and non-coding RNAs (e.g., miRNAs)—further modulate CD44v transcription and splicing. The upstream regulation of CD44v is orchestrated by a multilayered network encompassing signaling pathways, transcription factors, AS regulators, epigenetic modifications, and the tumor microenvironment. These mechanisms not only shape the expression landscape and isoform diversity of CD44v but also profoundly influence disease progression, including tumor initiation, invasion, metastasis, and therapeutic resistance. With the identification of novel regulatory circuits, such as the *SNORA37/CMTR1/ELAVL1* axis, our understanding of CD44v function in disease pathogenesis continues to expand. Targeting these upstream regulatory networks may offer novel avenues for precision diagnosis and therapeutic interventions.

## 6. Therapeutic Targeting of AS in Lung Cancer

AS events, often driven by alterations in the tumor microenvironment or genetic mutations that impair the function of splicing regulators such as SRSF1, RNA binding fox-1 homolog 1 (RBFOX1), and epithelial splicing regulatory protein (ESRP), play a pivotal role in promoting malignant transformation and augmenting oncogenic phenotypes, including increased metastatic potential and therapeutic resistance [62]. AS is a key factor in the onset of lung cancer, involving aberrant splicing events in genes such as epidermal growth factor receptor (*EGFR*), *KRAS*, tumor protein p53 (*TP53*, p53), *BCL2*, and *MET*. These aberrant splicing events play a crucial role in tumor behavior [63], treatment outcomes, and drug resistance. Substantial evidence from lung cancer research indicates that genome-wide dysregulation of AS is closely associated with tumorigenesis, metastasis, and therapeutic resistance. Drug resistance and metastasis constitute core impediments to lung cancer therapy, markedly attenuating the efficacy of targeted therapies and immunotherapies while accelerating disease progression and perpetuating a vicious prognostic cycle through epithelial–mesenchymal transition (EMT)-mediated tumor heterogeneity. However, the literature indicates that a critical DNA repair gene, Excision Repair Cross-Complementation Group 1 (*ERCC1*) exhibits splicing isoforms that not only modulate the capacity of lung cancer cells to repair cisplatin-induced DNA damage, but also influence CD3e molecule, epsilon associated protein (CD3EAP)-mediated proliferative signaling and protein phosphatase 1 regulatory subunit 13 like (PPP1R13L)-dependent suppression of p53 [12,30,34]. Meanwhile, previous studies have shown that AS isoforms of the ribosomal protein S24 (*RPS24*) gene, particularly the exon 4 to 22 base pairs microexon junction (ex4:22 bp) variant mediated by microexons, serve as molecular markers of cancer progression and EMT [35]. These isoforms exhibit tissue-specific expression patterns across multiple cancer types and modulate tumor invasion and metastatic potential through splicing factors such as RNA binding motif protein 47 (RBM47) [64]. With the continuous advancement of high-throughput RNA sequencing technologies, aberrant AS events in tumor cells are increasingly being identified. These molecular mechanisms are gaining attention for their roles in cancer initiation and progression, and they hold significant potential as novel cancer biomarkers [65]. Evidence suggests that splice-switching oligonucleotides (SSOs) can induce the expression of pro-apoptotic isoforms in cancer cells, leading to the inhibition of tumor growth [66], as demonstrated in both in vitro cell culture and xenograft models. Collectively, these findings highlight the pivotal role of AS events in driving lung cancer pathogenesis—from oncogenic activation and EMT-mediated tumor heterogeneity to therapeutic resistance—thereby positioning splice variants of key genes, such as *RPS24*, *ERCC1*, and as robust biomarkers for prognostic evaluation and therapeutic targeting [34,35]. Leveraging high-throughput RNA sequencing and SSO-based approaches offers transformative potential for precision oncology, underscoring the need for prospective studies to translate these insights into clinical strategies that attenuate metastasis and drug resistance [12,33]. The biological mechanisms of the abnormal AS isoforms of more genes in lung cancer and their functions are shown in Table 2.

## 7. Future Perspectives and Conclusions

### 7.1. Scope, Coverage, and Limitations

This review examines AS as a mechanistic determinant of lung cancer initiation, progression, metastasis, immune evasion, and treatment resistance. Representative oncogenic and tumor-suppressive splice variants are summarized, and these events are related to key splicing regulators and microenvironmental cues. Particular attention is given to subtype-specific splicing patterns in LUAD and LUSC, as well as to translational strategies that target AS. Rather than providing an exhaustive compendium of reported isoforms, this article offers a mechanistic and translational synthesis. Therefore, formal clinical-trial meta-analyses and comprehensive benchmarking of computational pipelines are outside its scope.

### 7.2. Critical View

Although aberrant AS has attracted growing attention in lung cancer, the supporting evidence remains heterogeneous in methodological rigor and translational relevance. Many isoform-phenotype relationships are derived predominantly from a limited set of experimental models and have not been validated consistently in well-annotated patient cohorts or systematically examined across LUAD and LUSC. This approach undermines reproducibility and limits the generalizability of biomarkers. Furthermore, AS is often interpreted as a downstream transcriptomic signature rather than as a causally structured regulatory network shaped by oncogenic signaling, microenvironmental pressures, and dynamic splicing-factor activity, leaving key mechanistic hierarchies unresolved. Therefore, a synthesis that considers subtypes and integrates mechanistic evidence with actionable translational hypotheses is needed to prioritize clinically relevant AS events, regulators, and therapeutic opportunities.

### 7.3. Future Perspectives

AS is increasingly recognized not as a mere epiphenomenon but as a fundamental oncogenic mechanism that can cooperate with canonical driver alterations to promote lung cancer development [72,73]. Distinct lung cancer subtypes exhibit unique splicing landscapes, and pervasive isoform switching can substantially reshape protein structure and function [72,74]. Across both LUAD and LUSC, aberrant AS events show pronounced differences between tumor and normal tissues and can modulate the function of multiple cancer-relevant genes, underscoring the potential of splicing signatures as molecular biomarkers and their contribution to cancer initiation and progression. Notably, splice alterations have been documented in established driver genes, including *TP53*, Rac family small GTPase 1 (*RAC1*)/RAC1b, erb-b2 receptor tyrosine kinase 2 (*ERBB2*), and neurotrophic receptor tyrosine kinase 2 (*NTRK2*), supporting a direct role for AS dysregulation in tumorigenesis. In addition, many splicing abnormalities occur independently of overt genetic lesions, suggesting that AS constitutes a regulatory layer parallel to genomic mutations. Consistent with this view, splicing dysregulation is closely associated with metastasis and therapeutic resistance [75]. For example, aberrant Cluster of Differentiation 19 (CD19) isoforms have been implicated in failure of CAR-T therapy.

Therapeutic strategies targeting splicing dysregulation are advancing rapidly. Current approaches include small-molecule inhibitors of the core spliceosome, kinase inhibitors that modulate splicing-relevant regulatory pathways, agents that promote the degradation of RNA-binding proteins (RBPs), antisense oligonucleotides that are engineered to correct pathogenic splicing, and synthetic intron-based systems that are designed to selectively eliminate cancer cells harboring spliceosomal mutations. In parallel, tumor-specific neoantigens arising from aberrant splicing may offer additional targets for immunotherapeutic intervention. Collectively, splicing dysregulation is not only a hallmark of cancer initiation and progression but also a potentially actionable vulnerability. Continued integration of advances in genomics, chemical biology, and synthetic biology is expected to expand the repertoire of precision cancer therapies [76].

Driven by rapid advances in high-throughput sequencing and computational biology [72,74], methods for detecting and functionally interrogating AS continue to mature. Third-generation long-read platforms (e.g., PacBio and Nanopore) enable direct sequencing of full-length transcripts, improving the resolution and accuracy of complex splicing-event identification [76]. Single-cell transcriptomics further delineates tumor heterogeneity and cell type-specific splicing programs within the tumor microenvironment, whereas spatial transcriptomics contextualizes splicing patterns within tissue architecture. Moreover, integrating deep learning-based splicing prediction, RNA structural analyses, and protein–protein interaction network modeling is accelerating a shift toward mechanistic, systems-level understanding of cancer-associated AS.

Lung cancer exhibits profound molecular and cellular heterogeneity, which continues to hinder mechanistic dissection and therapeutic development. CD44v warrant particular attention because of their emerging potential as biomarkers and therapeutic targets and their established roles in lung cancer biology [77,78]. However, CD44v isoforms display marked functional diversity, with distinct structural compositions and context-dependent biological effects across tumor types. Current CD44v research is limited by several key gaps. First, the regulatory network controlling CD44 pre-mRNA splicing remains incompletely defined, particularly with respect to the contributions of core splicing factors such as the SRSF family [67] and RNA-binding proteins such as QKI [68]. Second, lung cancer-focused studies are relatively sparse [69], and systematic comparisons of subtype-specific CD44v expression patterns and functional roles between LUAD and LUSC are lacking. Accordingly, in-depth mechanistic investigations of CD44v in lung cancer are needed. Future work should prioritize three directions: first, evaluating specific CD44v isoforms and their upstream splicing regulators as diagnostic and prognostic biomarkers by defining their roles in tumorigenesis, therapeutic resistance, and metastasis [70] in LUAD and LUSC; second, elucidating the mechanisms generating distinct CD44v isoforms, with emphasis on regulatory interactions between splicing factors and CD44 pre-mRNA; and third, testing rational combination strategies that target CD44v isoforms and/or their splicing control to provide robust preclinical evidence in support of personalized lung cancer therapy.

### 7.4. Conclusions

In conclusion, although the role of AS has been partially elucidated in other cancer types, its regulatory mechanisms and therapeutic relevance in lung cancer remain inadequately explored [71]. Addressing these gaps through focused mechanistic studies will be essential for the development of next-generation targeted therapies, particularly for tackling metastasis and drug resistance—two of the most pressing challenges in lung cancer treatment.

## Figures and Tables

**Table 1:** Comprehensive comparison of molecular, biological, and therapeutic differences between LUAD and LUSC.

Feature	LUAD	LUSC
Origin	Derived from distal airway epithelial cells, including alveolar type II cells and bronchiolar epithelial cells	Derived from proximal airway basal cells undergoing squamous differentiation
Dominant genomic alterations	EGFR mutation KRAS mutation ALK fusion ROS1 fusion BRAF V600E MET exon14 skipping/amplification	TP53 mutation PIK3CA mutation FGFR1 amplification SOX2 amplification CDKN2A loss KEAP1 mutation NFE2L2 mutation PTEN loss NOTCH pathway alterations
Epidemiological characteristics	The predominant NSCLC subtype, enriched in never-smokers, females, and East Asian populations, with a rising incidence and younger age distribution.	A smoking-associated NSCLC subtype with male predominance, originating mainly from proximal bronchial epithelium.
Genomic landscape	Enriched for actionable oncogenic drivers (EGFR, KRAS, ALK, ROS1, BRAF, MET)	Characterized by tumor suppressor alterations and genomic instability (TP53 mutation, CDKN2A loss, SOX2/FGFR1 amplification)
Key signaling pathways	EGFR/MAPK, PI3K-AKT, RAS and RTK signaling	Notch signaling, oxidative stress, cell-cycle regulation
Biological characteristics	Oncogenic driver-dependent proliferation and therapeutic vulnerability	Squamous differentiation, genomic instability, immune regulation
Alternative splicing landscape	Alternative splicing alterations influence RNA processing, epithelial–mesenchymal transition, proliferation and therapeutic resistance	Mainly involved in differentiation, adhesion, immune regulation and stress responses
Immune context	Immune heterogeneity influenced by driver alterations and oncogenic signaling	Higher tumor mutation burden and smoking-associated neoantigens contribute to immunotherapy responsiveness
Therapeutic implication	Multiple actionable genomic alterations enable precision medicine approaches	Immunotherapy remains a major therapeutic strategy, while molecularly targeted therapies are emerging for selected genomic alterations

**Table 2:** Function of key gene AS isoforms in lung cancer progression.

Disease Type	Gene and AS Isoforms	Role of AS Isoforms in Progression of the Disease	Reference
LUAD	*NUMB* and *NUMB-L/NUMB-S*	NUMB-L inhibits tumor migration; NUMB-S promotes EMT, migration and invasion	[15]
LUAD	*MAZ* and *MAZ-2*	Alter the KRAS regulatory network and participate in the carcinogenic process of lung cancer	[16]
LUSC	*FGFR1* and *FGFR1-β*	Up-regulate the MAPK/ERK pathway to drive mitosis and cell proliferation. It is related to the sensitivity of FGFR inhibitors	[37]
LUAD	*PARP4*	Loss or mutation of PARP4 disrupts its interaction with the splicing factor hnRNPM, leading to widespread intron retention and exon skipping events in genes involved in RNA metabolism, thereby promoting tumorigenicity	[49]
LUSC	*VEGFR1* and *sVEGFR1-i13*	By regulating the VEGF165-SOX2-SRSF2 signaling network, it reshapes the autocrine and integrin signaling pathways of tumor cells, promoting tumor progression and mediating resistance to anti-angiogenic therapy	[67]
LUAD + LUSC	*ADD3* and *ADD3 exon 14*	Promote the proliferation and migration of tumor cells by enhancing the functions related to the cytoskeleton	[68]
LUSC	*ARRB1* and *ARRB1-S*	Promote the proliferation, migration and invasion of tumor cells, thereby advancing the progression of LUSC	[69]
LUAD	*MET* and *METex14*	By avoiding CBL-mediated degradation → prolonging the MET half-life. Increasing carcinogenic signals and drive tumor growth	[70]
LUAD	*AXIN2* and *AXIN2-S*	By inhibiting the degradation of β-catenin and continuously activating the Wnt/β-catenin signaling pathway, it promotes EMT and induces chemotherapy and EGFR-TKI resistance	[71]

## Data Availability

This review article did not involve the generation or analysis of new datasets. All data discussed in this manuscript are available from the cited published literature.

## References

[1] Rahal Z., El Darzi R., Moghaddam S.J., Cascone T., Kadara H. (2025). Tumour and microenvironment crosstalk in NSCLC progression and response to therapy. Nat. Rev. Clin. Oncol..

[2] Yang X., Wu H. (2024). RAS signaling in carcinogenesis, cancer therapy and resistance mechanisms. J. Hematol. Oncol..

[3] Li H., Sun L., Gao P., Hu H. (2024). Lactylation in cancer: Current understanding and challenges. Cancer Cell.

[4] Zhang L., Xu J., Zhou S., Yao F., Zhang R., You W., Dai J., Yu K., Zhang Y., Baheti T. (2024). Endothelial DGKG promotes tumor angiogenesis and immune evasion in hepatocellular carcinoma. J. Hepatol..

[5] Wu Q., You L., Nepovimova E., Heger Z., Wu W., Kuca K., Adam V. (2022). Hypoxia-inducible factors: Master regulators of hypoxic tumor immune escape. J. Hematol. Oncol..

[6] Zhang G., Gao Z., Guo X., Ma R., Wang X., Zhou P., Li C., Tang Z., Zhao R., Gao P. (2023). CAP2 promotes gastric cancer metastasis by mediating the interaction between tumor cells and tumor-associated macrophages. J. Clin. Investig..

[7] Thai A.A., Solomon B.J., Sequist L.V., Gainor J.F., Heist R.S. (2021). Lung cancer. Lancet.

[8] Chen P., Liu Y., Wen Y., Zhou C. (2022). Non-small cell lung cancer in China. Cancer Commun..

[9] de Sousa V.M.L., Carvalho L. (2018). Heterogeneity in lung cancer. Pathobiology.

[10] Song X., Xiong A., Wu F., Li X., Wang J., Jiang T., Chen P., Zhang X., Zhao Z., Liu H. (2023). Spatial multi-omics revealed the impact of tumor ecosystem heterogeneity on immunotherapy efficacy in patients with advanced non-small cell lung cancer treated with bispecific antibody. J. Immunother. Cancer.

[11] Lim Z.F., Ma P.C. (2019). Emerging insights of tumor heterogeneity and drug resistance mechanisms in lung cancer targeted therapy. J. Hematol. Oncol..

[12] Sciarrillo R., Wojtuszkiewicz A., Assaraf Y.G., Jansen G., Kaspers G.J.L., Giovannetti E., Cloos J. (2020). The role of alternative splicing in cancer: From oncogenesis to drug resistance. Drug Resist. Updates.

[13] Han B., Park H.K., Ching T., Panneerselvam J., Wang H., Shen Y., Zhang J., Li L., Che R., Garmire L. (2017). Human DBR1 modulates the recycling of snRNPs to affect alternative RNA splicing and contributes to the suppression of cancer development. Oncogene.

[14] Alsafadi S., Dayot S., Tarin M., Houy A., Bellanger D., Cornella M., Wassef M., Waterfall J.J., Lehnert E., Roman-Roman S. (2021). Genetic alterations of *SUGP1* mimic mutant-*SF3B1* splice pattern in lung adenocarcinoma and other cancers. Oncogene.

[15] Bechara E.G., Sebestyén E., Bernardis I., Eyras E., Valcárcel J. (2013). RBM5, 6, and 10 differentially regulate NUMB alternative splicing to control cancer cell proliferation. Mol. Cell.

[16] Lo A., McSharry M., Berger A.H. (2022). Oncogenic KRAS alters splicing factor phosphorylation and alternative splicing in lung cancer. BMC Cancer.

[17] Das T., Lee E.-Y., You H.J., Kim E.E., Song E.J. (2022). USP15 and USP4 facilitate lung cancer cell proliferation by regulating the alternative splicing of SRSF1. Cell Death Discov..

[18] Zong F.-Y., Fu X., Wei W.-J., Luo Y.-G., Heiner M., Cao L.-J., Fang Z., Fang R., Lu D., Ji H. (2014). The RNA-binding protein QKI suppresses cancer-associated aberrant splicing. PLoS Genet..

[19] Alamgeer M., Watkins D.N., Banakh I., Kumar B., Gough D.J., Markman B., Ganju V. (2018). A phase IIa study of HA-irinotecan, formulation of hyaluronic acid and irinotecan targeting CD44 in extensive-stage small cell lung cancer. Investig. New Drugs.

[20] Coomer A.O., Black F., Greystoke A., Munkley J., Elliott D.J. (2019). Alternative splicing in lung cancer. Biochim. Biophys. Acta Gene Regul. Mech..

[21] Kargi H.A., Kuyucuoglu M.F., Alakavuklar M., Akpmar O., Erk S. (1997). CD44 expression in metastatic and non-metastatic non-small cell lung cancers. Cancer Lett..

[22] Todaro M., Gaggianesi M., Catalano V., Benfante A., Iovino F., Biffoni M., Apuzzo T., Sperduti I., Volpe S., Cocorullo G. (2014). CD44v6 is a marker of constitutive and reprogrammed cancer stem cells driving colon cancer metastasis. Cell Stem Cell.

[23] Chen C., Zhao S., Karnad A., Freeman J.W. (2018). The biology and role of CD44 in cancer progression: Therapeutic implications. J. Hematol. Oncol..

[24] Ni J., Cheung B.B., Beretov J., Duan W., Bucci J., Malouf D., Graham P., Li Y. (2020). CD44 variant 6 is associated with prostate cancer growth and chemo-/radiotherapy response in vivo. Exp. Cell Res..

[25] Gomez K.E., Wu F., Keysar S.B., Morton J.J., Miller B., Chimed T.-S., Le P.N., Nieto C., Chowdhury F.N., Tyagi A. (2020). Cancer cell CD44 mediates macrophage/monocyte-driven regulation of head and neck cancer stem cells. Cancer Res..

[26] Ishimoto T., Nagano O., Yae T., Tamada M., Motohara T., Oshima H., Oshima M., Ikeda T., Asaba R., Yagi H. (2011). CD44 variant regulates redox status in cancer cells by stabilizing the xCT subunit of system xc^−^ and thereby promotes tumor growth. Cancer Cell.

[27] Ariza A., Mate J.L., Isamat M., López D., Von Uexküll-Güldeband C., Rosell R., Fernández-Vasalo A., Navas-Palacios J.J. (1995). Standard and variant CD44 isoforms are commonly expressed in lung cancer of the non-small cell type but not of the small cell type. J. Pathol..

[28] Xu H., Niu M., Yuan X., Wu K., Liu A. (2020). CD44 as a tumor biomarker and therapeutic target. Exp. Hematol. Oncol..

[29] Yan Y., Zuo X., Wei D. (2015). Concise review: Emerging role of CD44 in cancer stem cells: A promising biomarker and therapeutic target. Stem Cells Transl. Med..

[30] Zhang G., Xue P., Cui S., Yu T., Xiao M., Zhang Q., Cai Y., Jin C., Yang J., Wu S. (2018). Different splicing isoforms of ERCC1 affect the expression of its overlapping genes CD3EAP and PPP1R13L, and indicate a potential application in non-small cell lung cancer treatment. Int. J. Oncol..

[31] de Miguel F.J., Pajares M.J., Martinez-Terroba E., Ajona D., Morales X., Sharma R.D., Pardo F.J., Rouzaut A., Rubio A., Montuenga L.M. (2016). A large-scale analysis of alternative splicing reveals a key role of QKI in lung cancer. Mol. Oncol..

[32] Blázquez-Encinas R., García-Vioque V., Caro-Cuenca T., Moreno-Montilla M.T., Mangili F., Alós-Pérez E., Ventura S., Herrera-Martínez A.D., Moreno-Casado P., Calzado M.A. (2023). Altered splicing machinery in lung carcinoids unveils NOVA1, PRPF8 and SRSF10 as novel candidates to understand tumor biology and expand biomarker discovery. J. Transl. Med..

[33] Wang Y., Chen H., Qian Y. (2014). RBM4-regulated alternative splicing suppresses tumorigenesis. Cancer Discov..

[34] Friboulet L., Olaussen K.A., Pignon J.P., Shepherd F.A., Tsao M.S., Graziano S.L., Kratzke R.A., Douillard J.Y., Seymour L., Popper R. (2013). ERCC1 Isoform Expression and DNA Repair in Non–Small-Cell Lung Cancer. N. Engl. J. Med..

[35] Park J., Nam D.H., Kim D., Chung Y.-J. (2024). RPS24 alternative splicing is a marker of cancer progression and epithelial–mesenchymal transition. Sci. Rep..

[36] Shen Y., Chen J.-Q., Li X.-P. (2025). Differences between lung adenocarcinoma and lung squamous cell carcinoma: Driver genes, therapeutic targets, and clinical efficacy. Genes Dis..

[37] Makinen N., Meyerson M. (2023). Genomic insights into the mechanisms of FGFR1 dependency in squamous cell lung cancer. J. Clin. Investig..

[38] Sato T., Yoo S., Kong R., Sinha A., Chandramani-Shivalingappa P., Patel A., Fridrikh M., Nagano O., Masuko T., Beasley M.B. (2019). Epigenomic profiling discovers trans-lineage SOX2 partnerships driving tumor heterogeneity in lung squamous cell carcinoma. Cancer Res..

[39] Li Y., Xie T., Wang S., Yang L., Hao X., Wang Y., Hu X., Wang L., Li J., Ying J. (2024). Mechanism exploration and model construction for small cell transformation in EGFR-mutant lung adenocarcinomas. Signal Transduct. Target. Ther..

[40] Shi Q., Xue C., Zeng Y., Yuan X., Chu Q., Jiang S., Wang J., Zhang Y., Zhu D., Li L. (2024). Notch signaling pathway in cancer: From mechanistic insights to targeted therapies. Signal Transduct. Target. Ther..

[41] Bao G., Li T., Guan X., Yao Y., Liang J., Xiang Y., Zhong X. (2022). Development of a prognostic alternative splicing signature associated with tumor microenvironment immune profiles in lung adenocarcinoma. Front. Oncol..

[42] Zhao Q., Caballero O.L., Davis I.D., Jonasch E., Tamboli P., Yung W.K.A., Weinstein J.N., Strausberg R.L., Yao J., Kenna Shaw for TCGA research network (2013). Tumor-specific isoform switch of the fibroblast growth factor receptor 2 underlies the mesenchymal and malignant phenotypes of clear cell renal cell carcinomas. Clin. Cancer Res..

[43] Liu Y., Jia W., Li J., Zhu H., Yu J. (2020). Identification of survival-associated alternative splicing signatures in lung squamous cell carcinoma. Front. Oncol..

[44] Ghatak S., Hascall V.C., Markwald R.R., Feghali-Bostwick C., Artlett C.M., Gooz M., Bogatkevich G.S., Atanelishvili I., Silver R.M., Wood J. (2017). Transforming growth factor beta1 (TGFβ1)-induced CD44V6-NOX4 signaling in pathogenesis of idiopathic pulmonary fibrosis. J. Biol. Chem..

[45] Jin M., Liu B., Chen C., Huang Y., Zhang H., Chen B., Song G., Zhao D., Duan L., Liu W. (2023). Genome-wide splicing quantitative expression locus analysis identifies causal risk variants for non-small cell lung cancer. Cancer Res..

[46] Liu Y., Zhang C., Zheng Y., Jin S., Niu J., Liu Z., Wu X., Feng Z., Hu X., Feng H. (2025). Transcriptomic analysis reveals lung cancer and subtype-specific alternative splicing biomarkers regulated by RNA-binding proteins. Mol. Ther. Nucleic Acids.

[47] Climente-González H., Porta-Pardo E., Godzik A., Eyras E. (2017). The functional impact of alternative splicing in cancer. Cell Rep..

[48] Yan J.Q., Liu M., Ma Y.L., Le K.D., Dong B., Li G.H. (2021). Development of alternative splicing signature in lung squamous cell carcinoma. Med. Oncol..

[49] Lee Y.F., Phua C.Z.J., Yuan J., Zhang B., Lee M.Y., Kannan S., Chiu Y.H.J., Koh C.W.Q., Yap C.K., Lim E.K.H. (2024). PARP4 interacts with hnRNPM to regulate splicing during lung cancer progression. Genome Med..

[50] Nian Q., Li Y., Li J., Zhao L., Rodrigues Lima F., Zeng J., Liu R., Ye Z. (2024). U2AF1 in various neoplastic diseases and relevant targeted therapies for malignant cancers with complex mutations. Oncol. Rep..

[51] Walter D.M., Cho K., Sivakumar S., Denney D., Lee I.T., Dohlman A.B., Heinz J.M., Shurberg E., Jiang K.X., Gupta A.A. (2026). U2AF1 mutations rescue deleterious exon skipping induced by KRAS mutations. Nat. Genet..

[52] Peng Z., Xie L., Zhou S., Li Y. (2025). Separase inhibition enhances gefitinib sensitivity of lung cancer via PTBP1/TAK1/RIPK1-mediated PANoptosis. MedComm.

[53] Peng L., Zhao Y., Tan J., Hou J., Jin X., Liu D.-X., Huang B., Lu J. (2024). PRMT1 promotes Warburg effect by regulating the PKM2/PKM1 ratio in non-small cell lung cancer. Cell Death Dis..

[54] Liao M., Li C., Yang R., Li J., Wu K., Zhang J., Zhu Q., Shi Y., Zhang X. (2025). HNRNPC promotes progression of non-small cell lung cancer by maintaining TFAP2A mRNA stability. Cancer Cell Int..

[55] Yan Y., Ren Y., Bao Y., Wang W. (2023). RNA splicing alterations in lung cancer pathogenesis and therapy. Cancer Pathog. Ther..

[56] Oka M., Xu L., Suzuki T., Yoshikawa T., Sakamoto H., Uemura H., Yoshizawa A.C., Suzuki Y., Nakatsura T., Ishihama Y. (2021). Aberrant splicing isoforms detected by full-length transcriptome sequencing as transcripts of potential neoantigens in non-small cell lung cancer. Genome Biol..

[57] Hassn Mesrati M., Syafruddin S.E., Mohtar M.A., Syahir A. (2021). CD44: A multifunctional mediator of cancer progression. Biomolecules.

[58] Liu Y.-N., Tsai M.-F., Wu S.-G., Chang T.-H., Shih J.-Y. (2024). CD44s and CD44v8–10 isoforms confer acquired resistance to osimertinib by activating the ErbB3/STAT3 signaling pathway. Life Sci..

[59] Batsche E., Yi J., Mauger O., Kornobis E., Hopkins B., Hanmer-Lloyd C., Muchardt C. (2021). CD44 alternative splicing senses intragenic DNA methylation in tumors via direct and indirect mechanisms. Nucleic Acids Res..

[60] Maltseva D., Tonevitsky A. (2023). RNA-binding proteins regulating the CD44 alternative splicing. Front. Mol. Biosci..

[61] Bao B., Tian M., Wang X., Yang C., Qu J., Zhou S., Cheng Y., Tong Q., Zheng L. (2025). SNORA37/CMTR1/ELAVL1 feedback loop drives gastric cancer progression via facilitating CD44 alternative splicing. J. Exp. Clin. Cancer Res..

[62] Zhang Y., Qian J., Gu C., Yang Y. (2021). Alternative splicing and cancer: A systematic review. Signal Transduct. Target. Ther..

[63] Sun Q., Han Y., He J., Wang J., Ma X., Ning Q., Zhao Q., Jin Q., Yang L., Li S. (2023). Long-read sequencing reveals the landscape of aberrant alternative splicing and novel therapeutic target in colorectal cancer. Genome Med..

[64] Du Y., Rokavec M., Hermeking H. (2026). RBM47-Induced Gasdermin A/GSDMA Mediates Mesenchymal–Epithelial Transition and Pyroptosis of Colorectal Cancer Cells. Cancers.

[65] Chen H., Tang J., Xiang J. (2025). Alternative splicing in tumorigenesis and cancer therapy. Biomolecules.

[66] Bauman J.A., Li S.-D., Yang A., Huang L., Kole R. (2010). Anti-tumor activity of splice-switching oligonucleotides. Nucleic Acids Res..

[67] Abou Faycal C., Gazzeri S., Eymin B. (2019). A VEGF-A/SOX2/SRSF2 network controls VEGFR1 pre-mRNA alternative splicing in lung carcinoma cells. Sci. Rep..

[68] Wang J.Z., Fu X., Fang Z., Liu H., Zong F.Y., Zhu H., Yu Y.-F., Zhang X.-Y., Wang Y.H., Hui J. (2021). QKI-5 regulates the alternative splicing of cytoskeletal gene *ADD3* in lung cancer. J. Mol. Cell Biol..

[69] Wu L., Zhang F., Chen H., Zhao G. (2025). LSM12 promotes lung squamous cell carcinoma progression through mediating alternative splicing of ARRB1. Commun. Biol..

[70] Ghosh P., Pecora I., Park M. (2025). Mechanistic insights into MET exon 14 skipping mutations and their role in tumor progression. Biochem. Soc. Trans..

[71] Ouyang J., Zhang Y., Hou X., Xiong F., Shi L., Zhong Y., Yang L., Guo C., Zeng Z., Yan Q. (2025). Long non-coding RNA AFAP1-AS1 promotes alternative splicing of AXIN2 by facilitating SRSFs phase separation to induce drug resistance in lung adenocarcinoma. Mol. Cancer.

[72] Luo W., Xu M., Wong N., Ng C.S. (2025). Alternative splicing in lung adenocarcinoma: From bench to bedside. Cancers.

[73] Bonner E.A., Lee S.C. (2023). Therapeutic targeting of RNA splicing in cancer. Genes.

[74] De Paoli-Iseppi R., Gleeson J., Clark M.B. (2021). Isoform age: Splice isoform profiling using long-read technologies. Front. Mol. Biosci..

[75] Niu Z., Xu B., Li W., Sun J., Liang H. (2025). RNA splicing: Novel star in pulmonary diseases with a treatment perspective. Acta Pharm. Sin. B.

[76] Suo Y., Song Y., Wang W., Liu Q., Rodriguez H., Zhou H. (2025). Advancements in proteogenomics for preclinical targeted cancer therapy research. Biophys. Rep..

[77] Wallach-Dayan S.B., Rubinstein A.M., Hand C., Breuer R., Naor D. (2008). DNA vaccination with CD44 variant isoform reduces mammary tumor local growth and lung metastasis. Mol. Cancer Ther..

[78] Guan H., Nagarkatti P.S., Nagarkatti M. (2011). CD44 reciprocally regulates the differentiation of encephalitogenic Th1/Th17 and Th2/regulatory T cells through epigenetic modulation involving DNA methylation of cytokine gene promoters, thereby controlling the development of experimental autoimmune encephalomyelitis. J. Immunol..

